# Pelvic angiography is effective for emergency pediatric patients with pelvic fractures: a propensity-score-matching study with a nationwide trauma registry in Japan

**DOI:** 10.1007/s00068-019-01154-w

**Published:** 2019-05-22

**Authors:** Yusuke Katayama, Tetsuhisa Kitamura, Tomoya Hirose, Takeyuki Kiguchi, Tasuku Matsuyama, Hiroki Takahashi, Kosuke Kiyohara, Junya Sado, Shingo Adachi, Tomohiro Noda, Junichi Izawa, Yuko Nakagawa, Takeshi Shimazu

**Affiliations:** 1 Department of Traumatology and Acute Critical Medicine, Osaka University Graduate School of Medicine, 2-15 Yamada-oka, Suita, 565-0871 Japan; 2 Division of Environmental Medicine and Population Sciences, Department of Social and Environmental Medicine, Osaka University Graduate School of Medicine, 2-15 Yamada-oka, Suita, Japan; 3 Emergency and Critical Care Center, Osaka Police Hospital, 10-31 Kitayama-cho, Tennoji-ku, Osaka, Japan; 4 Kyoto University Health Services, Yoshida-honmachi, Sakyo-ku, Kyoto, Japan; 5 Department of Emergency Medicine, Kyoto Prefectural University of Medicine, 465 Kajiicho, Hiroko-ji noboru, Kawaramachi-dori, Kamigyo-ku, Kyoto, Japan; 6 Department of Emergency and Critical Medicine, Kansai Medical University, 2-3-1 Shin-machi, Hirakata, Japan; 7 Department of Food Science, Faculty of Home Economics, Otsuma Women’s University, 12 Sanban-cho, Chiyoda-ku, Tokyo, Japan; 8 Department of Health and Sport Sciences, Medicine for Sports and Performing Arts, Osaka University Graduate School of Medicine, 2-15 Yamada-oka, Suita, Japan; 9 Rinku General Medical Center, Senshu Trauma and Critical Care Center, 2-23 Rinku Orai-kita, Izumisano, Japan; 10 Department of Traumatology and Critical Care Medicine, Osaka City University Graduate School of Medicine, 1-5-7 Asahi-machi, Abeno-ku, Osaka, Japan; 11 Intensive Care Unit, Department of Anesthesiology, The Jikei University School of Medicine, 3-19-18 Nishi-Shinbashi, Minato-ku, Tokyo, Japan

**Keywords:** Pelvic fracture, Angiography, Children, Shock, Propensity-score matching

## Abstract

**Purpose:**

The aim of this study was to evaluate the association between the implementation of pelvic angiography (PA) and outcome in emergency pediatric patients with pelvic fracture.

**Methods:**

We extracted data on pelvic fracture patients aged ≤ 19 years between 2004 and 2015 from a nationwide trauma registry in Japan. The main outcome was hospital mortality. We assessed the relationship between implementation of PA and hospital mortality using one-to-one propensity-score-matching analysis to reduce potential confounding effects in comparing the PA group with the non-PA group.

**Results:**

In total, 1351 patients were eligible for our analysis, with 221 patients (16.4%) included in the PA group and 1130 patients (83.6%) included in the non-PA group. For all patients, the proportion of hospital mortality was higher in the PA group than in the non-PA group [13.6% (30/221) vs 7.1% (80/1130), crude odds ratio (OR) 2.062 (95% confidence interval (CI), 1.318–3.224); *p *= 0.002]. In the propensity-score-matched patients, the proportion of hospital mortality was lower in the PA group than in the non-PA group [10.5% (22/200) vs 18.2% (38/200), *p *= 0.027]. This finding was confirmed in both the multivariable logistic regression model [adjusted OR 0.392 (95% CI, 0.171–0.896); *p *= 0.026] and the conditional logistic regression model [conditional OR 0.484 (95% CI, 0.261–0.896); *p *= 0.021].

**Conclusion:**

The implementation of PA was significantly associated with lower hospital mortality among emergency pediatric patients with pelvic fractures compared with the non-implementation of PA.

## Introduction

Pelvic fracture is one of the severe traumas associated with high mortality due to fatal bleeding. There are several treatment strategies such as external fixation, retroperitoneal pelvic packing (RPP), and transcatheter arterial embolization (TAE) plus pelvic angiography (PA). According to the Eastern Association for the Surgery of Trauma (EAST) guidelines, emergency PA is recommended for adult patients with pelvic fractures who show hemodynamic instability, signs of ongoing bleeding after non-pelvic sources of blood loss, or arterial intravenous contrast extravasation in the pelvis by enhanced computed tomography (CT) [1]. Indeed, Falzarano and colleagues reported that 16.5% (87/528) of pelvic fracture patients had hemodynamic instability, and 11.9% (63/528) of them received emergency angiography and embolization [2].

However, pelvic fractures are rarer among children than among adults; the incidence is between 2.4% and 7.5% [3–5]. It is difficult to select appropriate sheathes and catheters for pediatric patients with pelvic fractures, because the body size of children is smaller than that of adults. Therefore, angiography or arterial embolization for emergency pediatric patients with pelvic fractures is rarely performed. Although there were some case series and case reports on angiography and catheter embolization in these patients [6–10], the effectiveness of PA in them has not been extensively investigated.

The Japanese Trauma Data Bank (JTDB) is a nationwide hospital-based trauma registry in Japan [11]. Using this database, we evaluated the association between the implementation of PA and outcome among emergency pediatric patients with pelvic fractures, based on a propensity-score (PS)-matching method.

## Methods

### Study design, population, and setting

This study was a retrospective observational study using the JTDB database. The study period spanned 12 years from January 2004 to December 2015. We included emergency pediatric patients aged ≤ 19 years who had pelvic fractures, were then transported to JTDB-participating hospitals, and were registered in the database. We extracted pediatric patients with pelvic fractures based on the following Abbreviated Injury Scale (AIS) codes: 852,600.2, 852,602.2, 852,604.3, 852,606.4, 852,608.4, 852,610.5, 852,800.3, and 853,000.3. In addition, we excluded those who were in cardiopulmonary arrest on hospital arrival, underwent inter-hospital transport, and had missing information on factors such as vital signs on hospital arrival, sex, outcome, or Injury Severity Score (ISS). This study defined patients in cardiopulmonary arrest as those whose systolic blood pressure was 0 mmHg and/or heart rate was 0 bpm on hospital arrival. This study was approved by the ethics committee of Osaka University Graduate School of Medicine. Personal identifiers were removed beforehand from the JTDB database, and thus, the patients’ right to informed consent was waived. This manuscript was written based on the STROBE statement to assess the reporting of cohort and cross-sectional studies [12].

### Japanese trauma data bank

The JTDB was established by the Japanese Association for the Surgery and Trauma (Trauma Surgery Committee) and the Japanese Association for Acute Medicine (Committee for Clinical Care Evaluation) [13, 14] and is similar to trauma databases in North America, Europe, and Oceania [15]. By 2016, 256 major emergency medical institutions around Japan had been registered in the JTDB database [14]. These hospitals have an ability equal to Level I trauma centers in the United States. Data were collected via the Internet from participating institutions. In most cases, the physicians and technicians who attended an AIS coding course had registered the data [15].

The JTDB captures data on trauma patients that includes age, sex, mechanism of injury, AIS code (version 1998), ISS, vital signs on hospital arrival, date and some time series from hospital arrival to discharge, and medical treatments such as interventional radiology and CT scanning and complications in accordance with regular forms for coding items [16]. The ISS was calculated from the top three scores of the AIS for nine sites classified by the AIS code.

From the JTDB database, we extracted age, sex, time of day and day of the week of hospital admission, calendar year, shock on hospital arrival, mechanism of injury, ISS, existence of injury with AIS score of three or more for each site, and implementation of PA. This study defined daytime as 09:00 am to 17:59 pm and nighttime as 18:00 pm to 08:59 am and also defined shock as a systolic blood pressure below 80 mmHg on hospital arrival [17].

### Endpoint

The main endpoint was hospital mortality.

### Statistical analysis

Pre- and in-hospital information and outcome were evaluated between the PA group and the non-PA group. To reduce potential confounding effects in the comparisons between the two groups, we estimated a PS by fitting a logistic regression model that was adjusted for the following nine variables before the decision to conduct PA was made: age (continuous variable), sex (male or female), time of day (daytime, nighttime, unknown), day of the week (weekday or weekend), shock on hospital arrival (presence or absence), ISS (continuous variable), existence of injury with AIS score of three or more for each site (yes or no), mechanism of injury (car occupant, motorcycle, bicycle, pedestrian, fall from high place, other, and unknown), and calendar year (2004–2006, 2007–2009, 2010–2012, and 2013–2015). We performed a receiver operating characteristic curve analysis with an area under the curve of PS for predicting the implementation of PA among the patients with pelvic fractures. One-to-one pair matching between the PA group and the non-PA group was performed by nearest-neighbor matching without replacement, using calipers of width equal to 0.2 of the standard deviation mean differences (SMD) before and after matching. When the SMD was less than 0.25 [18], we considered this to indicate a negligible imbalance between the two groups.

We investigated the association between the implementation of PA and hospital mortality among pediatric patients with pelvic fractures using univariable, multivariable, and conditional logistic regression analyses. On the basis of these analyses, we calculated their odds ratios (ORs) with 95% confidential intervals (CIs). In the multivariable logistic regression model, we adjusted for the above nine variables used in the PS calculation. In addition, we performed a subgroup analysis by shock status on hospital arrival (presence or absence). All tests were two-tailed, and *p* values of < 0.05 were considered statistically significant. All statistical analyses were performed with the use of SPSS version 23.0 J (IBM Corp., Armonk, NY) and R version 3.1.0 (The R Foundation for Statistical Computing).

## Results

**Fig. 1:**
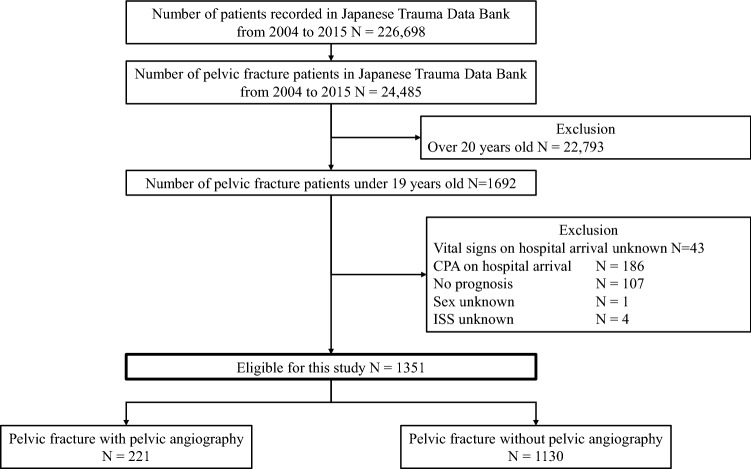
Flowchart of patient enrollment

Figure 1 shows the patient flow in this study. During the study period, 226,698 emergency patients were registered in the JTDB database, and 24,485 patients suffered pelvic fractures. Of them, 22,793 patients were ≥ 20 years, and 1692 patients were ≤ 19 years. After excluding 341 patients for the reasons shown in Fig. 1, 1351 patients were eligible for our analysis, with 221 patients (16.4%) included in the PA group and 1130 patients (83.6%) included in the non-PA group.

The clinical characteristics of the eligible patients in the PA group and the non-PA group before and after PS matching are shown in Table 1. Among all patients before PS matching, those in the PA group were more likely to be older, have shock on hospital arrival and a high ISS score, and to have received injuries to the abdomen and lower extremities including the pelvis with an AIS score of three or more compared with those in the non-PA group. As for the mechanism of injury, the proportion of patients with a fall from a high place was higher, but that of car occupant, bicycle, and pedestrian were lower in the PA group than in the non-PA group. After PS matching, 200 patients from each group were selected, and the area under the receiver operating characteristic curve of the PS was 0.823. The covariate balance between the two groups of matched patients improved greatly.

**Table 1:** Patient characteristics among all cohorts and propensity-score-matched cohort

	All patients			Propensity-score-matched patients		
	Pelvic angiography (+)	Pelvic angiography (−)	SMD	Pelvic angiography (+)	Pelvic angiography (−)	SMD
	(*N* = 221)	(*N* = 1130)		(*N* = 200)	(*N* = 200)	
Age, median (IQR)	17 (15–19)	16 (13–18)	0.260	17 (15–19)	17 (15–18)	0.000
Male, *n* (%)	124 (56.1)	684 (60.5)	0.090	112 (56.0)	124 (62.0)	0.120
Time of the day, *n* (%)						
Daytime (9:00 am—5:59 pm)	84 (32.1)	458 (40.5)	0.050	77 (38.5)	81 (40.5)	0.040
Nighttime (6:00 pm—8:59 am)	127 (60.6)	614 (54.3)	0.060	114 (57.0)	113 (56.5)	0.010
Unknown	10 (5.0)	58 (5.1)	0.030	9 (4.5)	6 (3.0)	0.080
Weekend, n (%)	48 (21.7)	334 (29.6)	0.180	46 (23.0)	45 (22.5)	0.010
Shock on hospital arrival, n (%)	46 (18.1)	74 (6.5)	0.420	36 (18.0)	34 (17.0)	0.030
ISS, median (IQR)	30 (21–41)	18 (10–29)	0.830	29 (20–38)	28 (17–42)	0.040
Number of patients with AIS ≥ 3 by body region, n (%)						
Head	61 (27.6)	320 (28.3)	0.020	51 (25.5)	54 (27.0)	0.030
Face	4 (1.8)	12 (1.1)	0.060	4 (2.0)	5 (2.5)	0.030
Thorax	115 (52.0)	496 (43.9)	0.160	98 (49.0)	96 (48.0)	0.020
Abdomen	66 (29.9)	115 (10.2)	0.510	52 (26.0)	51 (25.5)	0.010
Spine	19 (8.6)	73 (6.5)	0.080	16 (8.0)	18 (9.0)	0.040
Upper extremity	18 (8.1)	78 (6.9)	0.050	15 (7.5)	20 (10.0)	0.090
Lower extremity including pelvis	186 (84.2)	500 (44.2)	0.920	166 (83.0)	164 (82.0)	0.030
Unspecified	1 (0.1)	1 (0.1)	0.070	1 (0.5)	1 (0.5)	0.000
Mechanism of injury, n (%)						
Car occupants	15 (6.8)	131 (11.6)	0.170	15 (7.5)	15 (7.5)	0.000
Motorcycle	61 (27.6)	275 (24.3)	0.070	55 (27.5)	58 (29.0)	0.030
Bicycle	26 (11.8)	179 (15.8)	0.120	26 (13.0)	32 (16.0)	0.090
Pedestrian	22 (10.0)	186 (16.5)	0.190	20 (10.0)	14 (7.0)	0.110
Fall from high place	79 (35.7)	283 (25.0)	0.230	66 (33.0)	69 (34.5)	0.030
Other	16 (7.2)	62 (5.5)	0.070	16 (8.0)	12 (6.0)	0.080
Unknown	3 (1.4)	19 (1.7)	0.030	3 (1.5)	1 (0.5)	0.100
Calendar year, n (%)						
2004–2006	22 (10.0)	87 (7.7)	0.080	19 (9.5)	21 (10.5)	0.030
2007–2009	52 (23.5)	241 (21.3)	0.050	47 (23.5)	48 (24.0)	0.010
2010–2012	57 (25.8)	400 (35.4)	0.210	54 (27.0)	54 (27.0)	0.000
2013–2015	90 (40.7)	402 (35.6)	0.110	80 (40.0)	77 (38.5)	0.030

The area under the receiver operating characteristic curve of the logistic regression model to calculate a propensity score was 0.823

*IQR* interquartile range, *ISS* injury severity score, *AIS* abbreviated injury scale, *SMD* standard mean difference

Table 2 shows the relationship between the implementation of PA and hospital mortality among the pediatric emergency patients with pelvic fractures. Among all patients before PS matching, the proportion of hospital mortality was higher in the patients in the PA group than in those in the non-PA group [13.6% (30/221) vs 7.1% (80/1130), crude OR 2.062 (95% CI 1.318–3.224); *p *= 0.002]. However, the PA group tended to have a low OR for hospital mortality in the multivariable logistic regression model [adjusted OR 0.684 (95% CI 0.346–1.354); *p *= 0.276]. In the PS matched patients, hospital mortality was lower in the PA group than in the non-PA group [10.5% (22/200) vs 18.2% (38/200), crude OR 0.527 (95% CI 0.299–0.929); *p *= 0.027], and there were statistically significant differences between the PA group and the non-PA group in both the multivariable logistic regression model [adjusted OR 0.392 (95% CI 0.171–0.896); *p *= 0.026] and the conditional logistic regression model [conditional OR 0.484 (95% CI 0.261–0.896); *p *= 0.021].

**Table 2:** Outcomes of pediatric pelvic fracture patients with or without pelvic angiography

	Total		With pelvic angiography		Without pelvic angiography		Crude OR	(95% CI)	Adjusted OR	(95% CI)	Conditional OR	(95% CI)
All patients*	(*N* = 1351)		(*N* = 221)		(*N* = 1130)							
Death at hospital discharge	110	(8.1%)	30	(13.6%)	80	(7.1%)	2.062	(1.318–3.224)	0.684	(0.346–1.354)	–	–
Propensity-score-matched patients*	(*N* = 400)		(*N* = 200)		(*N* = 200)							
Death at hospital discharge	60	(14.4%)	22	(10.5%)	38	(18.2%)	0.527	(0.299–0.929)	0.392	(0.171–0.896)	0.484	(0.261–0.896)

ORs were calculated for pediatric pelvic fracture patients with vs without pelvic angiography

*OR* odds ratio, *CI* confidence interval

*Adjusted for age, sex, time of the day, day of the week, ISS, AIS score by each body region, shock at hospital arrival, mechanism of injury, and of calendar year group

Hospital mortality between the two groups of the pediatric patients with pelvic fractures by shock status on hospital arrival is shown in Table 3. Among the patients without shock on hospital arrival, hospital mortality was higher in the PA group than in the non-PA group [9.2% (16/174) vs 4.7% (49/1049), crude OR 2.067 (95% CI 1.147–3.724)]. However, among the patients with shock on hospital arrival, t hospital mortality was lower in the PA group than in the non-PA group [30.4% (14/46) vs 39.2% (29/74), OR 0.679 (95% CI 0.310–1.485)], and the *p* for interaction was 0.026.

**Table 3:** Subgroup analysis of death at hospital discharge among pediatric pelvic fractures patients with or without pelvic angiography

	Pelvic angiography (+)		Pelvic angiography (−)		Crude OR	(95% CI)	*P* Value for interaction
	% (*n*/*N*)		% (*n*/*N*)				
Shock on hospital arrival*							0.026
Shock (−)	9.2	(16/174)	4.7	(49/1049)	2.067	(1.147–3.724)	
Shock (+)	30.4	(14/46)	39.2	(29/74)	0.679	(0.310–1.485)	

*OR* odds ratio, *CI* confidence interval

*Systolic blood pressure ≤ 80 mmHg

## Discussion

From the data of a nationwide trauma registry in Japan, this study showed that the implementation of PA was significantly associated with lower hospital mortality among emergency pediatric patients aged ≤ 19 years with pelvic fractures compared with non-implementation of PA. This result from a matched cohort after PS matching was almost similar to that from the entire patient cohort before PS matching, which reinforced the robustness of our results. This study showing the effectiveness of the angiographic assessment of pediatric patients with pelvic fractures provides important clues for discussing treatment strategies in these patients.

This study revealed that the implementation of PA was associated with a low proportion of hospital mortality among emergency pediatric patients with pelvic fractures. As the ability of CT scanning has continued to advance, its role has become important in the initial assessment of emergency trauma patients. Especially, extravasation confirmed by enhanced CT scanning is effective for detecting arterial bleeding, and PA is recommended for pelvic fracture patients with extravasation confirmed by enhanced CT scanning in the EAST guidelines [1]. However, some studies reported that emergency patients with abdominal traumas and/or pelvic fractures could be conservatively managed if their conditions were stable, even if there was extravasation on enhanced CT scanning [19–21]. Conversely, in another report, even if no extravasation was apparent on enhanced CT scanning, TAE for arterial bleeding confirmed by angiography was conducted and subsequently led to an improvement of patient outcome [21]. Thus, making a decision about treatment strategies such as embolization and surgery based solely on enhanced CT scanning might not be appropriate emergency care. Although we did not obtain information on extravasation on enhanced CT scanning, the present study suggested that the evaluation of arterial bleeding with the use of angiography in pediatric patients with pelvic fractures was effective regardless of the findings on enhanced CT scanning.

As with adult patients with pelvic fractures, some studies reported that external fixation and both RPP and TAE were also effective for pediatric patients [8, 9, 22, 23]. Angiography allows the detection and simultaneous treatment of arterial bleeding, and its implementation would be more effective for children. Recently, CT-guided surgery was reported for tile type C pelvic injury [24]. As the blood flow in children is less than that in adults, bloodless treatments such as CT-guided surgery and embolization might be effective for pediatric patients with pelvic fracture. Although it is difficult to select an appropriate sheath and/or catheter size for pediatric patients when angiography is performed, according to case studies of TAE performed in children with hepatic injury, splenic injury or pelvic fracture, TAE could be performed without complications such as vessel laceration or thrombosis [25–27]. Thus, if operators can select an appropriate catheter size for pediatric patients, angiography could be performed safely without complications.

In the subgroup analysis, hospital mortality among the pediatric pelvic fracture patients with shock on hospital arrival was lower in the PA group than in the non-PA group. In adult patients with pelvic fractures, some studies pointed out that various factors such as hemodynamic instability need to be considered before performing emergency angiography [28–32]. Therefore, among patients with shock on hospital arrival, the identification of arterial bleeding by proactive PA would lead to the simultaneous implementation of TAE and, subsequently, a better outcome. However, hospital mortality among those without shock on hospital arrival was higher in the PA group than in the non-PA group. Although the reasons for the implementation of PA were unclear in this study, the PA group might have had severe conditions for which arterial bleeding was suspected, and/or the non-PA group might have had minor pelvic fractures that did not require PA.

## Limitations

This study has several limitations. First, it assessed the relationship between PA and hospital mortality among pediatric patients with pelvic fractures but did not assess the reasons for the implementation of PA such as extravasation on enhanced CT scanning. Second, as there was also no information recorded on external fixation or RPP or on the type of pelvic fractures based on the AO/OTA classification [33] in the JTDB database, the influence of these factors on patient outcome was unknown. Third, it was unclear whether the cause of death was due to bleeding or other reasons such as traumatic brain injury and infection. Fourth, although nine variables were used to calculate the PS, there may be other factors that affect the implementation of PA. For example, factors such as hospital treatment protocols and decision-making by surgeons may affect the implementation of PA, but these data were not available in the JTDB database. However, the area under the receiver operating characteristic curve of PS was 0.823 in this model, which would sufficiently explain the implementation of PA. Finally, because this study was a retrospective observational study, there might be unknown confounding factors that influence the relationship between PA and hospital mortality.

## Conclusion

In this patient population, the implementation of PA was significantly associated with lower hospital mortality among emergency pediatric patients with pelvic fractures compared with the non-implementation of PA.

## Data Availability

The data that support the findings of this study are available from the JTDB, but the availability of these data is restricted.
